# Characterization of HAK protein family in *Casuarina equisetifolia* and the positive regulatory role of *CeqHAK6* and *CeqHAK11* genes in response to salt tolerance

**DOI:** 10.3389/fpls.2022.1084337

**Published:** 2023-02-03

**Authors:** Yujiao Wang, Yong Zhang, Yongcheng Wei, Jingxiang Meng, Chonglu Zhong, Chunjie Fan

**Affiliations:** ^1^ State Key Laboratory of Tree Genetics and Breeding, Chinese Academy of Forestry, Beijing, China; ^2^ Key Laboratory of State Forestry and Grassland Administration on Tropical Forestry, Academy of Forestry, Guangzhou, China; ^3^ Specialty Cash Crop Research Laboratory, Cotton Research Institute of Anhui Academy of Agricultural Sciences, Hefei, China

**Keywords:** *Casuarina equisetifolia*, *HAK/KUP/KT* transporter, K+ uptake, ROS, salt tolerance, *CeqHAK6*, *CeqHAK11*

## Abstract

The potassium transporter group of the *HAK/KUP/KT* (high-affinity K^+^)/*KUP* (K^+^ uptake)/*KT* (K^+^ transporter) family plays a crucial role in plant growth and development as well as in environmental adaptation such as tolerance to salt stress. HAK/KUP/KT genes and their functions have been characterized for a number of plant species, but they remain unknown for *Casuarina equisetifolia*, an important tree species for coastal protection in southern China and many other countries. In this study, 25 *HAK* genes were identified in the *C. equisetifolia* genome. Their gene structure, conserved motif, phylogeny, and expression were comprehensively and systematically analyzed to understand their functions. All *HAK* genes were relatively conserved and could be divided into four clusters. The expression level of two particular genes, *CeqHAK11* and *CeqHAK6*, increased significantly with the duration of salt treatment. To further elucidated their function in response to salt stress, subcellular localization, and their functional analysis were developed. Results revealed that *CeqHAK11* and *CeqHAK6* were localized on the plasma membrane, which mainly mediated high-affinity K^+^ uptake. Overexpression of *CeqHAK6* or *CeqHAK11* in *Arabidopsis* showed higher germination and survival rates and longer root length than wild-type (WT) under salt stress, suggesting that both genes improve tolerance to salt stress. Moreover, *CeqHAK6* and *CeqHAK11* improved their ability to tolerate salt stress by increasing the K^+^/Na^+^ ratio and antioxidant enzyme activities (CAT, POD, and SOD), and decreasing reactive oxygen species (ROS) accumulation. Consequently, *CeqHAK6* and *CeqHAK11* were verified as potassium transport proteins and could be applied for further molecular breeding for salt tolerance in *C. equisetifolia* or other crops to increasing salt tolerance.

## Introduction

Plant growth and crop productivity are hampered by salt stress, which is a major global environmental concern. The damage caused by salt stress to plants includes osmotic stress, ion toxicity, and secondary stresses such as oxidative damage triggered by osmotic stress and ion toxicity (Parida and Das, 2005; Munns and Tester, 2008). Potassium (K^+^) is an activator of many intracellular enzymes and occupies an important position in plant growth and development. Due to the similar physical properties of Na^+^ and K^+^, excessive accumulation of Na^+^ will produce ion toxicity to plants on the one hand, and inhibit the absorption of K^+^, resulting in nutrient deficiency in plants (Grabov, 2007). For plant salt tolerance, it may be more important to maintain a low Na^+^/K^+^ ratio relative to the absolute Na^+^ content (Wu et al., 2018).

The HAK/KUP/KT protein family (high-affinity K^+^/K^+^ uptake proteins/K^+^ transporter) is the largest family of potassium ion transporter proteins in plants, and plays a key role in mediating potassium ion transport (Wang and Wu, 2013; Li et al., 2018). To date, many *HAK*/*KUP*/*KT* genes have been identified and characterized in a variety of plant species. For instance, 13, 27, 21, and 31 *HAK* genes were identified in *Arabidopsis*, rice, tea, and poplar, respectively (Ahn et al., 2004; Gupta et al., 2008; He et al., 2012; Yang et al., 2020). Most HAK proteins contain 10-15 transmembrane structures, generally with a cytoplasmic loop from the second transmembrane region to the third transmembrane region, and the C-terminus of HAK proteins is longer than the N-terminus (Rubio et al., 2000; Banuelos et al., 2002; Gupta et al., 2008; Nieves-Cordones et al., 2016). Additionally, the HAK/KUP/KT family was divided into four clusters based on a phylogenetic tree analysis (Rubio et al., 2000; Gupta et al., 2008).

Until now, many *HAK* genes were characterized. *AtHAK5* is currently the most studied HAK family member in *Arabidopsis*, and the expression level of *AtHAK5* is significantly increased under potassium deficiency conditions. Seed germination and root growth of *Arabidopsis* were markedly inhibited under low potassium conditions further suggesting that *AtHAK5* mediates high-affinity K^+^ uptake and influences seed germination and growth development. (Ahn et al., 2004; Gierth et al., 2005; Rubio et al., 2008; Pyo et al., 2010). In another study, the knockout of *OsHAK16* was also found to inhibit the root growth and the net absorption rate of K^+^ under low K^+^ levels. Conversely, overexpression of *OsHAK16* in rice improved total K^+^ uptake and growth of transgenic plants, indicating that *OsHAK16* could improve the salt tolerance of rice by regulating K^+^ absorption and transport (Feng et al., 2019). Similarly, *HbHAK1* exhibited high K^+^ uptake activity under extremely low external K^+^ conditions and reduced Na^+^ toxicity under high salt stress (Zhang et al., 2020). *SiHAK1* was localized at the cell membrane and had been implicated in the HAK uptake activity in yeast and *Arabidopsis athak5* mutant plants (Zhang et al., 2018). Recently, *SlHAK20* gene was also found to enhance salt tolerance of the tomato plants (Wang et al., 2020). Given these, HAK proteins maintain K^+^/Na^+^ homeostasis by mediating K^+^ uptake and transport, which play an important role in enhancing plant salt tolerance.

*Casuarina equisetifolia* is a typical coastal tree species widely distributed in the tropics and subtropics, which widely used for fuel wood and paper industry. In addition, the bark of *C. equisetifolia* contains tannins, phenolic components, and antioxidant substances, which can be used as industrial or pharmaceutical raw materials (Aher et al., 2009).With the characteristics of salinity tolerance, wind resistance and coastal sand stabilization, *C. equisetifolia* has become the most important tree species in the coastal areas of southern China (Zhong et al., 2010). Its high salt tolerance provides an important material for characterizing key salt response genes and elucidate long-term salt tolerance of woody plant. Completing of genome sequencing will provide a basis for further research and breeding for salt resistance mechanism in this important species.

In the present study, 25 putative genes of the HAK family were identified from *C. equisetifolia* and a systematical analysis of this family was carried out. Notably, the expression of *CeqHAK11* and *CeqHAK6* increased with the duration of salt treatment and reached a maximum at 168 h by RNA-seq data. The expression levels of *CeqHAK6* and *CeqHAK11* in different tissues and salt stress were performed using qRT-PCR. Moreover, functional analysis of *CeqHAK6* and *CeqHAK11* heterologous expression yeast and *Arabidopsis*, which served as positive regulators in salt tolerance of plants, were investigated. The results of this study will provide a valuable reference for the further study of HAK proteins and also gene resources for the genetic improvement of salt tolerance in *C. equisetifolia* and other crops.

## Materials and methods

### Plant materials, growth conditions, and stress treatments

For the expression experiment, rooted cuttings of *C. equisetifolia* clone A8 were cultivated in pots containing vermiculite and black soil, and grown in a growth chamber for 8 weeks. For salt treatments, the 200mM NaCl solution was poured over the culture medium vermiculite and black soil, and shoots were harvested at 0, 1, 6, 24, and 168 h after treatment. Different tissues (root, stem, phloem, xylem, inflorescence, young and mature shoot) were taken from a 10-year-old tree of clone A8 in our nursery during the peak growing season (May) of *C. equisetifolia*. The bark surface (brown) of the trunk was scraped off with a one-sided blade and the tissue (light yellow) was taken as the phloem. A portion of the tissue was then scraped off with the blade to expose the white part of the tissue which was the xylem. Three biological replicates were set for each treatment.

Seeds of tobacco (*Nicotiana Tabacum L.*) and *Arabidopsis* (Col-0) were placed in Murashige and Skoog (MS) medium for three days at 4°C, and then transferred to a plant climate incubator (MLR-352H, Shanghai, China) with 16/8 h of light/dark at 22°C and 80% humidity. After ten days, they were transplanted to square pots (7 cm diameter) comprising a mixture of peat soil and vermiculite (1:3).

### Database search for HAKs in Casuarina equisetifolia

The whole-genome protein sequence of *C. equisetifolia* was downloaded from the online website (http://forestry.fafu.edu.cn/db/Casuarinaceae/) as a local database. The HAK protein sequences of poplar, *Arabidopsis* and rice were downloaded from the Phytozome database (https://phytozome.jgi.doe.gov/pz/portal.htmL). Local Blast (E value-5) searching was performed using the Hidden Markov Model (HMM) profile of HAK domains (PF02705) as queries for the identification of HAK genes from *C. equisetifolia*. Then, all candidate genes were manually deduplicated, and the candidate genes were verified to have HAK domains using Pfam, SMART, and NCBI online tools. Physical and chemical characterization of the identified HAK proteins was performed using the online tool ExPASy. Finally, the protein sequences of the identified members of the HAK protein family were submitted to the TMHMM website (http://www.cbs.dtu.dk/services/TMHMM-2.0/) and the PSORT website (http://www.psort.org/) to predict the number of transmembrane regions as well as the subcellular localization of the CeqHAK proteins.

### Bioinformatic analysis of the HAK gene family in *Casuarina equisetifolia*

Multiple sequence alignment of HAK full-length protein sequences from *C. equisetifolia*, poplar, *Arabidopsis*, and rice were performed using ClustalX 2.11. Based on the alignment, a comprehensive phylogenetic tree of all HAK proteins was constructed using MEGA 7.0 to classify and analyze the HAK gene family in *C. equisetifolia*.

Paralogous and orthologous pairs were identified according to the method of Blanc and Wolfe (2004). Moreover, the synonymous substitution (Ks) value and the non-synonymous substitution (Ka) value of the identified paralogous pairs were calculated by TBtools software.

The GFF (General feature format) files of *CeqHAK* genes were uploaded to the GSDS website (http://gsds.cbi.pku.edu.cn/) for analysis structure of exons and introns. To predict the conserved motifs of HAK gene in *C. equisetifolia*, 25 CeqHAK protein sequences were uploaded to the MEME website. The number of conserved motifs to be searched was set to 20, the length of the conserved motifs was between 6 and 200 amino acids, and other parameters were set to default.

Based on the transcriptome sequencing data of *C. equisetifolia*, the expression data of the members of the *CeqHAK* genes in response to salt stress were obtained, and the expression pattern heat map was completed using TBtools software (Chen et al., 2020). GO analysis was performed using the online website agriGO database, and a corrected *p* ≤ 0.05 threshold was used to screen significantly enriched GO terms.

### RNA extraction and expression analysis of *CeqHAKs*

Total RNA from *C. equisetifolia* samples was isolated using the Aidlab plant RNA kit (Aidlab Biotech, Beijing, China). Then, cDNA was synthesized using the Revert Aid First Strand cDNA Synthesis Kit (ThermoFisher Scientific, USA) according to the specification. Specific primers for *CeqHAK* genes (*CeqHAK6*-Q and *CeqHAK11*-Q) were designed by TBtools software and the *CeqEF1α* was used as a reference gene (**Table S1**). qRT-PCR was performed on an LightCycler480 II Real-Time PCR system (made in Switzerland) using TB Green Premix Ex Taq II (TaKaRa Biotechnology, Dalian, China), as described previously (Wang et al., 2021b). Each experiment was repeated three times, and the relative expression data results were calculated by 2^−ΔΔCT^.

### Isolation of the full-length *CeqHAK6* and *CeqHAK11*

Primers were designed for PCR amplification based on the coding sequences of *CeqHAK6* and *CeqHAK11* (**Table S1**). The amplification reaction was performed using PrimeSTAR Max DNA Polymerase (TaKaRa Biotechnology, Dalian, China) with the following amplification procedure: 98°C for 10 min, 40 cycles of 98°C for 10 s, 59°C for 30 s and 72°C for 20 s, and 72°C for 10 min. The PCR products were clone into the pEASY^®^-Blunt Cloning Vector, and positive clones were obtained by sequenced (Sangon, Shanghai, China).

### Subcellular localization analysis

The coding sequences of *CeqHAK6* and *CeqHAK11* (without the stop codon) were amplified by PCR using the primers *CeqHAK6*-Sub and *CeqHAK11*-Sub (**Table S1**). The PCR fragments were constructed into the pCAMBIA1305 vector (Clontech, Beijing, China) with green fluorescent protein (GFP) gene driven by the cauliflower mosaic virus (CaMV) 35S promoter, positive recombinant plasmid p1305-CaMV35S-*CeqHAK6*-GFP and p1305-CaMV35S-*CeqHAK11*-GFP were obtained after sequencing verification. The recombinant plasmids were transformed to the *Agrobacterium tumefaciens* strain EHA105. The suspension containing recombinant plasmid and plasma membrane marker gene (*OsNrat1*) (Xia et al., 2010) was injected into the lower epidermis of tobacco leaves and labeled. After two days in dark culture, GFP fluorescence was observed by confocal microscope (LSM710, Carl Zeiss, Jena, Germany).

### Functional analysis of *CeqHAK6* and *CeqHAK11* in yeast strain

PCR amplification of *CeqHAK6* and *CeqHAK11* cDNA using primers *CeqHAK6*-P2 and *CeqHAK11*-P2 (**Table S1**), and PCR products were inserted into the pYSE2 vector (ThermoFisher Scientific, USA) and used to transform R5421 (MATα ura3-52 leu2 trk1Δ his3Δ200 his4-15 trk2Δ1::pCK64) and BY4741 (MATa his3Δ1 leu2 met15Δ ura3-52) strain (Weidi Biotechnology, Shanghai, China). Transformants were screened by SD-Ura agar plates, and successfully transformed yeast cells were incubated in SD-Ura liquid medium at 30°C overnight until the optical density (OD)_600_ reached 0.7, and then serially diluted 10-fold on AP plates containing different contents of Na^+^ and K^+^. Finally, they were cultured in a constant temperature incubator at 30°C for 5-7 days and photographed for observation.

### Salt tolerance analysis of *CeqHAK6* and *CeqHAK11* in transgenic Arabidopsis

The primer *CeqHAK6*-OE and *CeqHAK11*-OE was designed according to the sequences of *CeqHAK6*, *CeqHAK11* and the pCAMBIA1300 vector (**Table S1**). The coding sequence of *CeqHAK6* and *CeqHAK11* were subcloned into pCAMBIA1300 vector driven by the CaMV35S promoter. and confirmed by sequencing. Recombinant plasmid p1300-CaMV35S-*CeqHAK6* and p1300-CaMV35S-*CeqHAK11* were confirmed by sequencing and subsequently transformed into *Arabidopsis* Col-0 by the floral dip method using *Agrobacterium tumefaciens* GV3101. Screening of first-generation transgenic *Arabidopsis* seeds (T_0_) on 1/2 MS medium containing 30 mg/L kanamycin and identification of transgenic plants by PCR and qPCR. Homozygous plants of the T_3_ transgenic lines were used for subsequent analysis.

For seed germination assay, T_3_ transgenic *Arabidopsis* seeds and wild-type (WT) seeds were sterilized and washed individually, then dispersed onto 1/2 MS plates with different salt concentration gradients (0 mM, 150 mM, and 175 mM NaCl), and the experiment and reference groups were duplicated three times, respectively. After 10 days, seed germination and survival rates were examined and determined. Germination rate = number of germinated seeds at the end of germination/number of tested seeds × 100%. Survival rate = number of germinated seeds and growth at the end of germination/number of tested seeds × 100%.

For root growth assay, T_3_ transgenic *Arabidopsis* seeds and WT seeds were sterilized and washed individually, then spreaded on 1/2 MS plates for germination. After a period of five days, three sprouted seedlings of the same height from each overexpression (OE) line were transplanted onto 1/2 MS media containing 0 or 150 mM NaCl. The length of the tap root of each seedling was measured after growing for seven days. The seedlings were placed in the medium, the taproot were straightened with tweezers, and the length was measured one by one.

For the salt treatment assay, seven-day old germinated transgenic and WT lines were grown in 7-cm square pots containing a mixture of peat soil and vermiculite (1:3). After 14 days, seedlings with similar growth stage were irrigated with 250 mM NaCl solution for ten days.

### Determination of ion content after salt treatment

The leaves of WT and transgenic *Arabidopsis* plants were treated with 250 mM NaCl solution were collected at 0, 1, 6, 24 and 168 h, respectively. A pot of four plants was used as one replicate, and the mean value of the data was calculated through three independent replicates. The dried leaves (80°C, 6 hours) were ground into powder in liquid nitrogen and then digested with concentrated HNO_3_ and ion (Na^+^ and K^+^) content analysed as described above (Wang et al., 2021a).

### Antioxidant enzyme activities and histochemical staining

The activities of catalase (CAT), peroxidase (POD), and superoxide dismutase (SOD) and the level of malondialdehyde (MDA) content, and hydrogen peroxide (H_2_O_2)_ content were determined by test kits from Solarbio (Guangzhou, China). Specific assays were performed following the instructions provied.

Ten days after salt treatment, three leaves of per lines were submerged in nitro blue tetrazolium (NBT) and 3,3’-diaminobenzidine (DAB) staining solution, wrapped in tin foil and placed in a vacuum pump for 30 min, and then stained at 37°C for 6 h for DAB and 4 h for NBT. After staining, the leaves were placed in a new 2 mL centrifuge tube containing 75% alcohol and heated in a metal bath at 75°C until the leaves were decolorized, and finally the leaves were taken out and imaging for observation.

### Statistical analysis

All data subjected to one-way analysis of variance in IBM SPSS Statistics 25 (IBM Corporation, Armonk, NY, USA) and significant differences between treatment means were determined using Duncan’s Multiple Range Test (*p*<0.05). The means and standard deviations (SDs) values of all experimental data were calculated from three biological replicates.

## Results

### A total of 25 *HAK* genes were identified in *C. equisetifolia*

After BLAST and HMM search and manual de-duplication, a total of 25 *HAK* genes in *C. equisetifolia* were obtained, namely *CeqHAK1* to *CeqHAK25* respectively. The sequence lengths of open reading frames (ORFs), scaffold positions, amino acid (aa) lengths, protein molecular weights (MW) and protein isoelectric points (PI) were subsequently collected using the ExPASy online website (**Table S2**). The longest amino acid length of *CeqHAK* genes was 849 aa and the shortest was 101 aa. Meanwhile, scaffold_2829 had 5 genes, scaffold_45 had 6 genes, and scaffold_41, scaffold_75 and scaffold_85 each had 2 genes on them. Most *CeqHAK* genes located in the plasma membrane by subcellular localization prediction (**Table S3**). Analysis of transmembrane structures of all CeqHAK proteins showed that *CeqHAK17* had 13 transmembrane structures while *CeqHAK22* and *CeqHAK23* only contained 2 transmembrane structures. These *CeqHAK* genes were likely related to ion transport and had different transport activities.

### Various gene and protein structure of CeqHAK proteins implied their function differences

**Figure 1:**
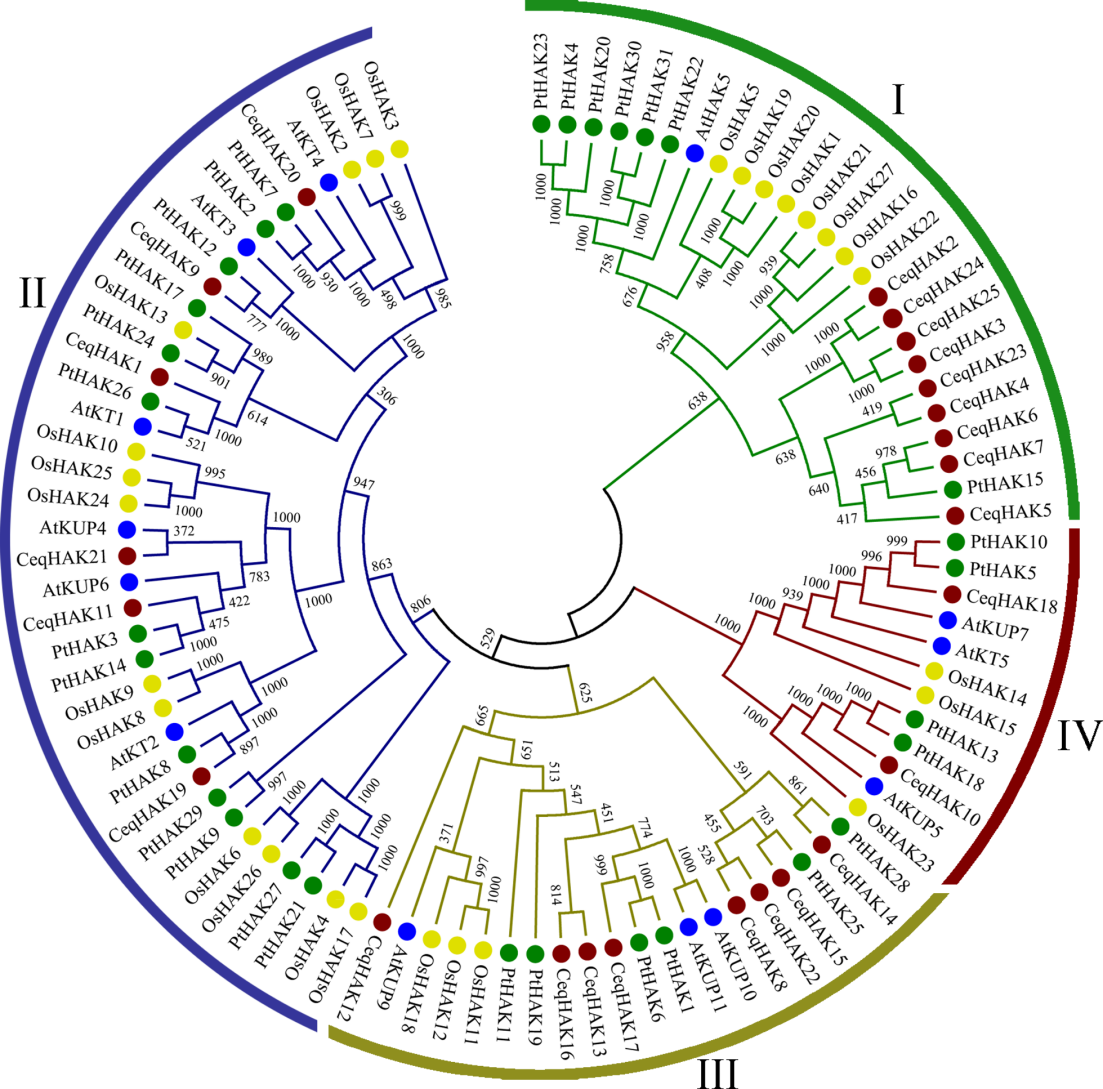
Phylogenetic tree of *HAK* genes from *C. equisetifolia*, *Arabidopsis*, rice and poplar. 25 *CeqHAK* genes, 13 *AtHAK* genes, 27 *OsHAK* genes and 31 *PtHAK* genes were clustered into four subgroups (I, II, III and IV). Red, blue, yellow and green shapes represent *HAK* genes from *C. equisetifolia*, *Arabidopsis*, rice, and poplar, respectively. The tree was generated with the MEGA 7.0 using the neighbor-joining (N-J) method.

Firstly, an unrooted phylogenetic tree was constructed using 13, 27, 31, and 25 HAK proteins from *Arabidopsis*, rice, poplar and *C. equisetifolia*, respectively (**Table S4**). *CeqHAKs* could be divided into four clusters, namely I, II, III, and IV (**Figure 1**), in which the cluster IV had two members only, while the other three clusters consisted of six to nine members. Moreover, based on bidirectional best-hit analysis, we identified 19 putative paralogous pairs wre identified in *C. equisetifolia* (**Table S5**), seven orthologous pairs in *C. equisetifolia* and *Arabidopsis*, 11 orthologous pairs in *C. equisetifolia* and rice, and 20 orthologous pairs in *C. equisetifolia* and poplar (**Table S6**). The Ka/Ks ratio of 17 paralogous pairs ranged from 0.091 to 0.490, indicating that *HAK* gene families in *C. equisetifolia* were mainly experienced purifying selection.

**Figure 2:**
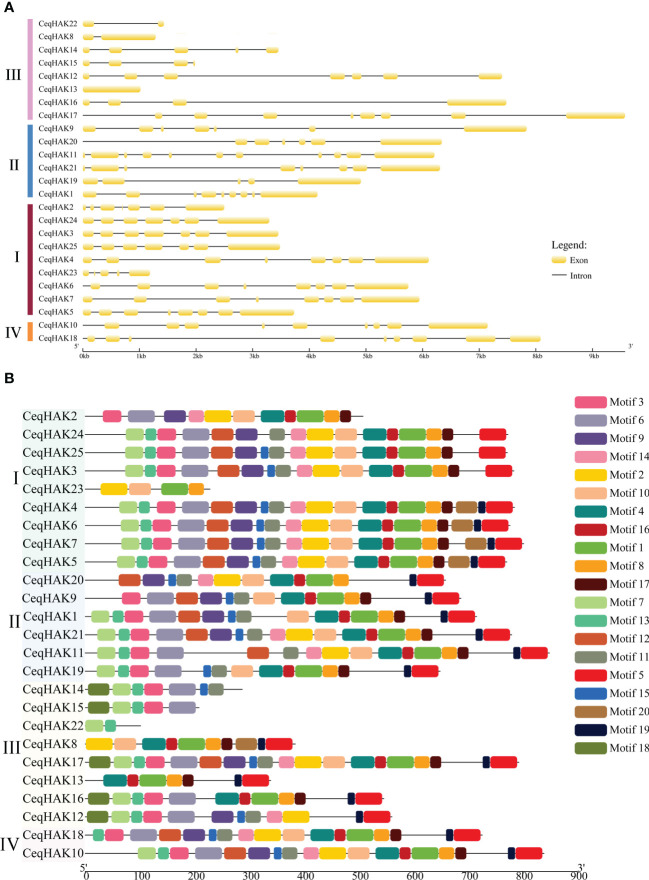
Gene structure and conserved motifs of *HAK* genes in *C equisetifolia*. **(A)** Gene structure analysis of *CeqHAKs*. Introns were depicted by black lines, and coding sequences were represented by yellow boxes. **(B)** Conserved motifs analysis of *CeqHAKs*. In different colored boxes, conservative motif numbers 1-20 are displayed.

Intron/exon structure maps were constructed to further understand the structural similarity and diversity of *CeqHAK* genes (**Figure 2A**). The structure of *CeqHAK* genes was highly variable, with the number of exons ranging from one to 11. Members in clusters I, II, and IV had the same or similar number and positions of exons while the exon numbers in cluster III ranged from one to eight. In addition, nine out of the 17 paralogous pairs had the same number of exons. However, the exon number of two gene pairs, *CeqHAK19*/*11* and *CeqHAK13*/*17*, was clearly different from that of the other paralogous pairs.

There were 20 distinct motifs in total (**Figure 2B**). The conserved amino acid sequences, lengths and annotation within each motif are presented **Table S7**. Thirteen of the 20 motifs were found to encode the K^+^ potassium transporter domain. Moreover, all *CeqHAK* genes had at least two conserved motifs of K_trans. Most of the paralogous pairs had the same or similar motif structures, especially for *CeqHAK3*/*24*, *CeqHAK5*/*6*, *CeqHAK6*/*7*, *CeqHAK6*/*4* and *CeqHAK24*/*25*. Members in same cluster had similar motif features, which further supported the phylogenetic classification of the *CeqHAK* gene family. In addition, the result of GO analysis indicated that all 25 *CeqHAK* genes were involved in potassium ion transport (**Table S8**).

### *CeqHAK6* and *CeqHAK11* continuously responded to salt stress

Based on the RNA-seq data of *CeqHAK* genes at different treatment times under 200 mM NaCl stress in the roots (Wang et al., 2021a), and 19 *CeqHAK* genes were induced by salt stress (**Figure S1** and **Table S9**). It was found that *CeqHAK25*, *CeqHAK12* and *CeqHAK7* shared a similar expression pattern, which was significantly up-regulated at 1 h of salt treatment, and then decreased with increasing treatment duration. Meanwhile, *CeqHAK20* and *CeqHAK21* expressions increased significantly at 6 h or 24 h of treatment. Notably, the expression levels of *CeqHAK11* and *CeqHAK6* increased with the duration of salt treatment and reached the maximum at 168 h, which implied that *CeqHAK11* and *CeqHAK6* play an important role in response to salt stress in *C. equisetifolia*.

**Figure 3:**
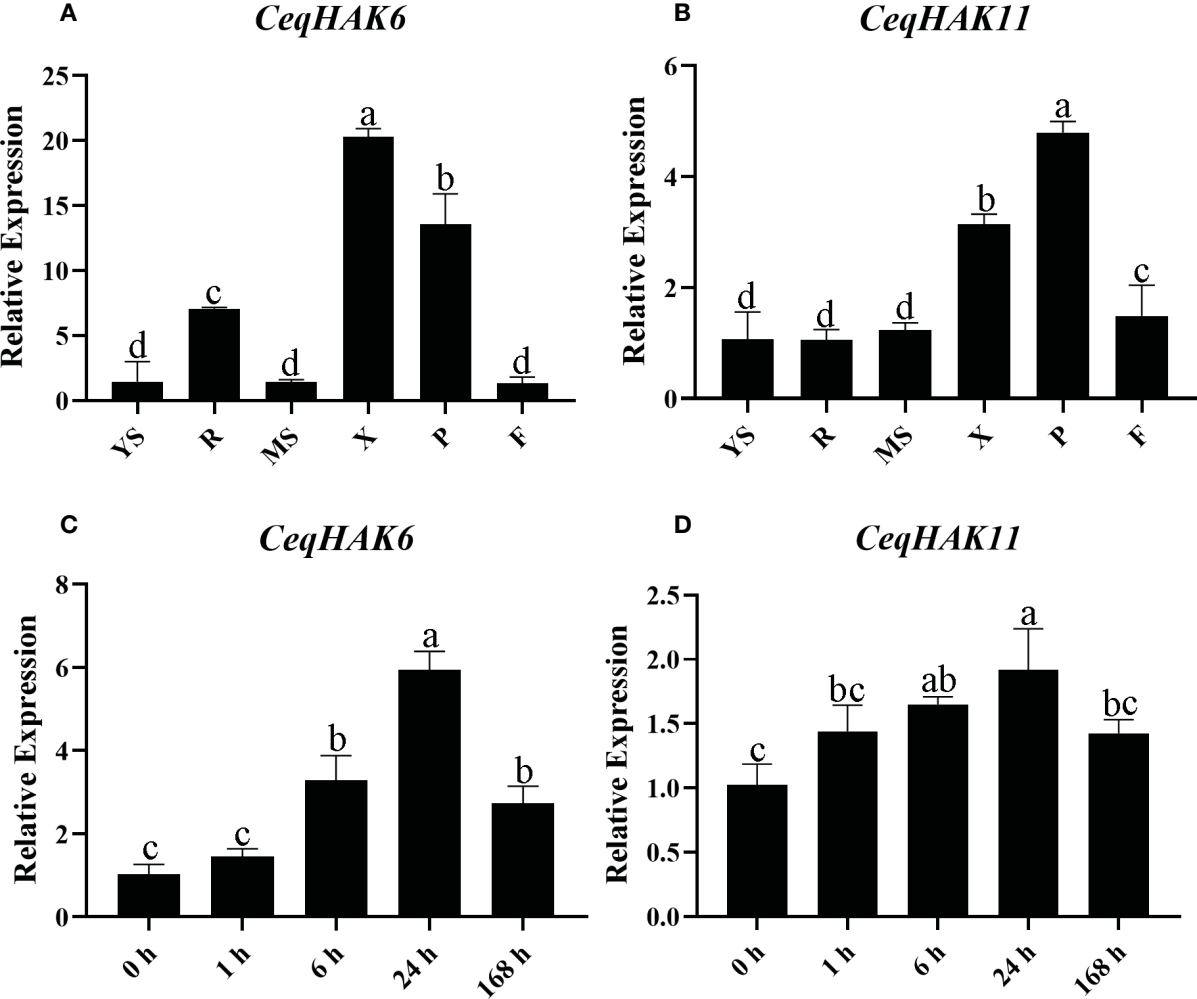
Expression analysis of *CeqHAK6* and *CeqHAK11* genes by qRT-PCR. **(A, B)** Relative expression of the *CeqHAK6* and *CeqHAK11* genes in different tissues of *C equisetifolia*. Data of other *C equisetifolia* tissues were normalized with the young shoot. YS, Young shoot; R, Root; MS, Mature shoot; X, Xylem of stem; P, Phloem of stem; F, Inforescence. **(C, D)** Relative expression of the *CeqHAK6* and *CeqHAK11* genes at different time under NaCl treatment of *C equisetifolia*. The expression level of *CeqEF1α* was used for data normalization. The bars represented the mean ± standard deviation (n = 3). The significant differences identified using Duncan’s Multiple Range Test (*p*<0.05) were denoted by different lowercase letters on the top of bars.

To further validate the response of *CeqHAK6* and *CeqHAK11* genes to salt stress, their expression in different tissues and salt treatments was detected by qPCR (**Figure 3**). *CeqHAK6* was highly expressed in the root and xylem and phloem of the stem while *CeqHAK11* was highly expressed in the phloem. Furthermore, both *CeqHAK6* and *CeqHAK11* genes showed a similar expression trend in shoots under salt stress. The expression increased with the extension of salt treatment time to attain the peaked at 24 h, followed by a slightly down-regulation at 168 h.

Following the cloning of *CeqHAK6* and *CeqHAK11* genes, the ORF length of *CeqHAK6* was 2334 bp and encoded 777 amino acids, while the ORF length of *CeqHAK11* was 2550 bp and encoded 849 amino acids (**Figure S2**). Sequence alignment analysis of the protein sequences of *AtKT5*, *OsHAK25*, *CeqHAK6* and *CeqHAK11* showed that Domain I (GXXXGDXXXSPLY) and Domain II (QXXALGCFPKXKIVHTSXKXXGQIYIPENWILM) were all present in the *HAK* genes (**Figure S3**).

### *CeqHAK6* and *CeqHAK11* localized on plasma membrane and mediated K^+^ uptake

**Figure 4:**
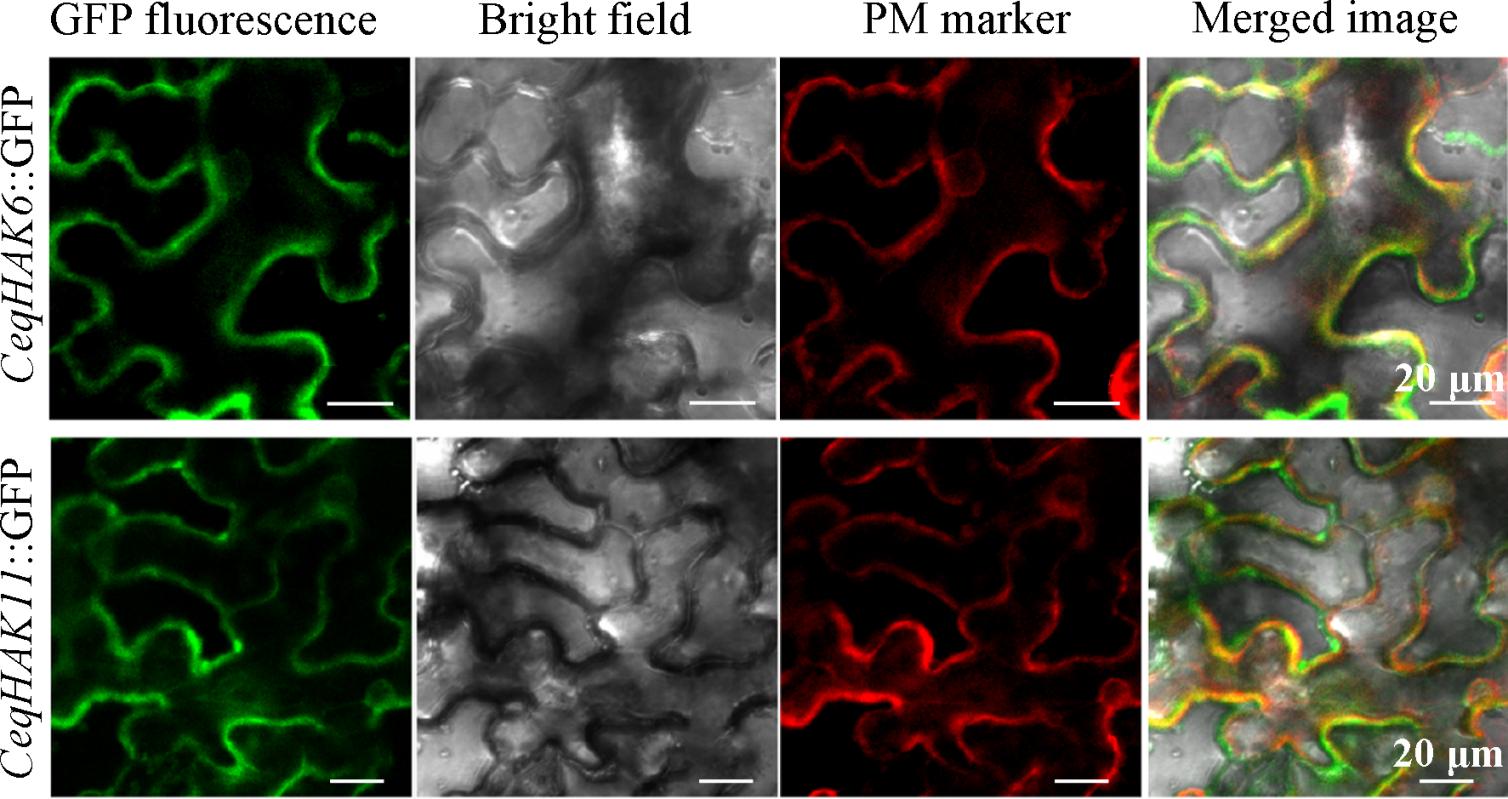
Subcellular location of CeqHAK6 and CeqHAK11 proteins. The fusion construct (*CeqHAK6*::GFP or *CeqHAK11*::GFP) and PM protein were transformed in tobacco leaves. Confocal microscopy images of tobacco leaf epidermal cells.

As shown in **Figure 4**, the proteins of *CeqHAK6* and *CeqHAK11* were located on the plasma membrane, which sat in the same position of PM marker protein located on the plasma membrane. These results were consistent with the prediction of subcellular localization.

**Figure 5:**
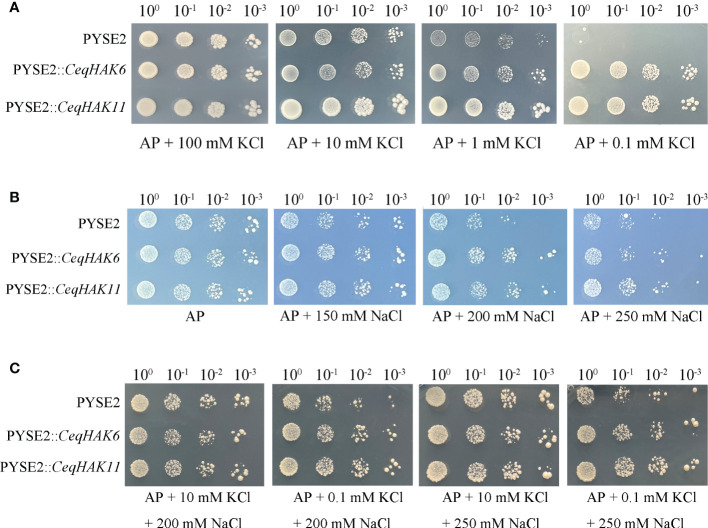
Functional characterization of *CeqHAK6* and *CeqHAK11* expressed in yeast strain. **(A)** Growth inhibition tests of yeast strain R5421 (MATα ura3-52 leu2 trk1Δ his3Δ200 his4-15 trk2Δ1::pCK64) expressed the *CeqHAK6* gene, *CeqHAK11* gene and empty vector pYES2. **(B, C)** Growth of yeast strain BY4741 (MATa his3Δ1 leu2 met15Δ ura3-52) cells harboring the *CeqHAK6* gene, *CeqHAK11* gene and empty vector pYES2. All transformants were grown on AP medium with different contents of Na^+^ and K^+^. Three independent experiments were performed.

To identify the hypothesis of K^+^ uptake by candidate genes, the vector of *CeqHAK6*-pYSE2, and *CeqHAK11*-pYSE2 were constructed and K^+^ transport activity was tested in yeast strain R5421. As shown in **Figure 5A**, the yeast strain carried *CeqHAK6* or *CeqHAK11* grew well and did not show clear differences from the control in AP medium with 100 mM K^+^. However, with decreasing K^+^ content in AP medium, the control yeast strain displayed a stronger inhibition of growth and was unable to grow in the medium containing 0.1 mM K^+^ while the yeast strain harboring *CeqHAK6* and *CeqHAK11* could grow well. It was also noted that *CeqHAK11*-expressing strain grew better than *CeqHAK6*-expressing strain. Both *CeqHAK6* and *CeqHAK11* appeared to have strong K^+^ transport activity, especially under extremely low-K^+^ conditions. Meanwhile, it concluded that CeqHAK*11* had a stronger ability to stimulate K^+^ uptake compared to *CeqHAK6*.

With regard to salt tolerance function, the yeast strain carriying *CeqHAK6* and *CeqHAK11* grew better than the control in medium containing 200 mM Na^+^(**Figure 5B**). To verify the alleviating sodium toxic function of *CeqHAK6* and *CeqHAK11* by K^+^ transport, K^+^ was supplemented in AP medium containing 200 mM and 250 mM Na^+^. The inhibition of growth under high salt was obviously alleviated in all yeast strain under the external 10 mM K^+^ condition (**Figure 5C**). The yeast strain carriying *CeqHAK6* and *CeqHAK11* grew well in medium containing 200 mM Na^+^ by supplying 10 to 0.1 mM K^+^ while the growth of the control was significantly inhibited. Similar results were observed when 250 mM Na^+^ was used. These results demonstrated that *CeqHAK6* and *CeqHAK11* could enhance the salt resistance by K^+^ transporting.

### Overexpression of *CeqHAK6* and *CeqHAK11* could improve salt tolerance in *Arabidopsis*

To elucidate the function of *CeqHAK6* and *CeqHAK11*, the expression vectors of 35S::*CeqHAK6* and 35S::*CeqHAK11* were constructed and then transformed into *Arabidopsis*. Undergoing selection and PCR checking, 10 lines of overexpression *CeqHAK6* and *CeqHAK11* were verified individually and further selected for analyzing by qRT-PCR analysis (**Figure S4**). The lines OE5, OE12 and OE13(*CeqHAK6*) and OE11, OE13 and OE17 (*CeqHAK11*) were selected for further phenotype analysis.

**Figure 6:**
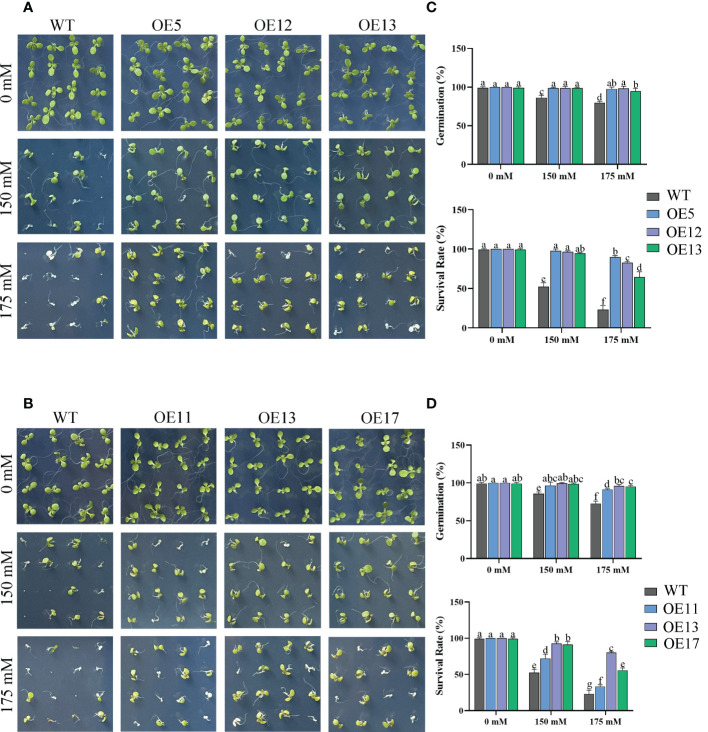
Germination of *CeqHAK6* and *CeqHAK11* transgenic lines under salt stress. **(A)** Germination of *CeqHAK6* transgenic lines under salt stress. **(B)** Germination of *CeqHAK11* transgenic lines under salt stress. Transgenic *Arabidopsis* seeds and WT seeds were spotted onto 1/2 MS plates with 0, 150, or 175 mM NaCl. **(C)** Calculation of the germination rates and survival rate of *CeqHAK6*-OE and WT seeds. **(D)** Calculation of the germination rates and survival rate of *CeqHAK11*-OE and WT seeds. The significant differences identified using Duncan’s Multiple Range Test (*p*<0.05) were denoted by different lowercase letters on the top of bars.

**Figure 7:**
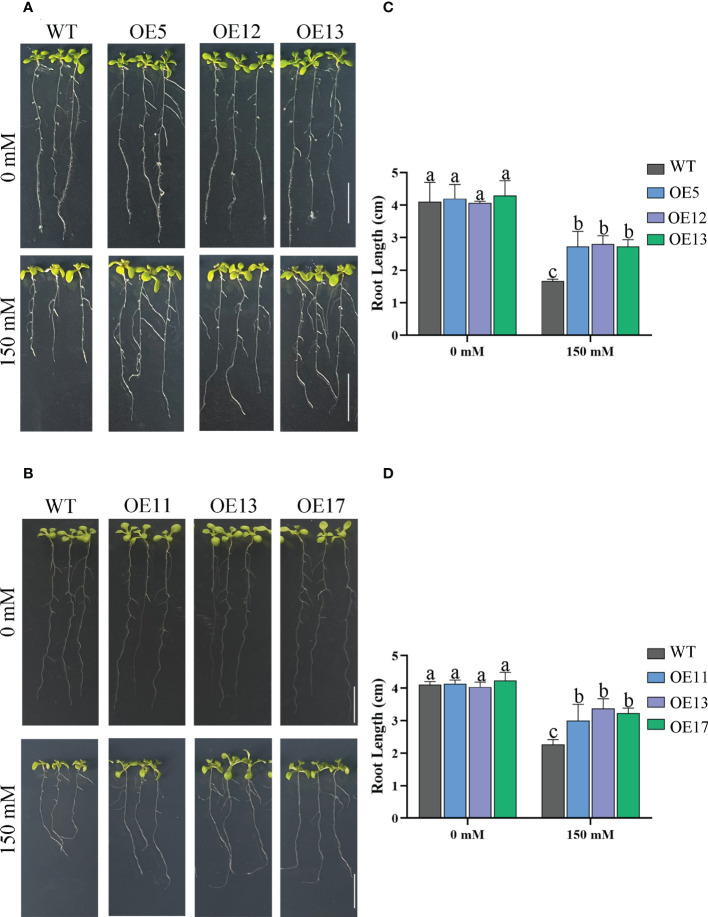
Primary root length of plants grown on media under salt treatment. **(A)** The root length of *CeqHAK6*-OE lines and WT plants under salt stress. **(B)** The root length of *CeqHAK11*-OE lines and WT plants under salt stress. Each seedling planted to 1/2 MS media containing 0 and 150 mM NaCl. Scale bar = 1 cm. **(C)** Calculation of the root length of *CeqHAK6* transgenic and WT plants. **(D)** Calculation of the root length of *CeqHAK11* transgenic and WT plants. The significant differences identified using Duncan’s Multiple Range Test (*p*<0.05) were denoted by different lowercase letters on the top of bars.

Seed germination affected by salt treatments. The germination rate and survival rates of WT seeds decreased markedly, especially under 175 mM salt treatment with only 23% survival (**Figure 6**). The germination rate of OE lines was not considerably reduced by 175 mM salt treatment. The survival rate of OE lines was also higher than that of WT. Similarly, the tap roots of transgenic lines were longer than those of WT plants under 150 mM NaCl treatment (**Figure 7**).

**Figure 8:**
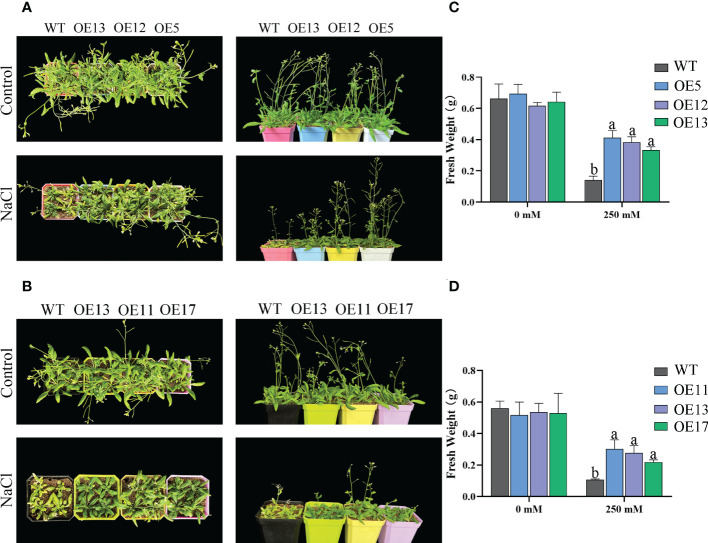
Improved salt tolerance in transgenic *Arabidopsis* plants expressing *CeqHAK6* and *CeqHAK11*. **(A)** Performance of *CeqHAK6* transgenic and WT plants before and after salt treatment. **(B)** Performance of *CeqHAK11* transgenic and WT plants before and after salt treatment. All Arabidopsis seedlings were treated with 250 mM NaCl for 10 days. **(C)** Fresh weight of *CeqHAK6* transgenic and WT plants before and after salt treatment. **(D)** Fresh weight of *CeqHAK11* transgenic and WT plants before and after salt treatment. The significant differences identified using Duncan’s Multiple Range Test (*p*<0.05) were denoted by different lowercase letters on the top of bars.

WT and the lines of overexpression *CeqHAK6* and *CeqHAK11* did not show obvious differences in the absence of salt treatment (**Figure 8**). Under 250 mM treatment, the lines of overexpression of *CeqHAK6* and *CeqHAK11* could maintain normal growth while WT growth was inhibited with wrinkled and yellowed leaves, fewer stems and wilted inflorescences. The fresh weight of the transgenic plants was significantly greater than that of the WT. In addition, OE5 lines showed better performance under salt stress compared to that of the other OE lines.

### *CeqHAK6* and *CeqHAK11* improved salt tolerance by increasing antioxidant enzyme activities and reducing ROS accumulation

**Figure 9:**
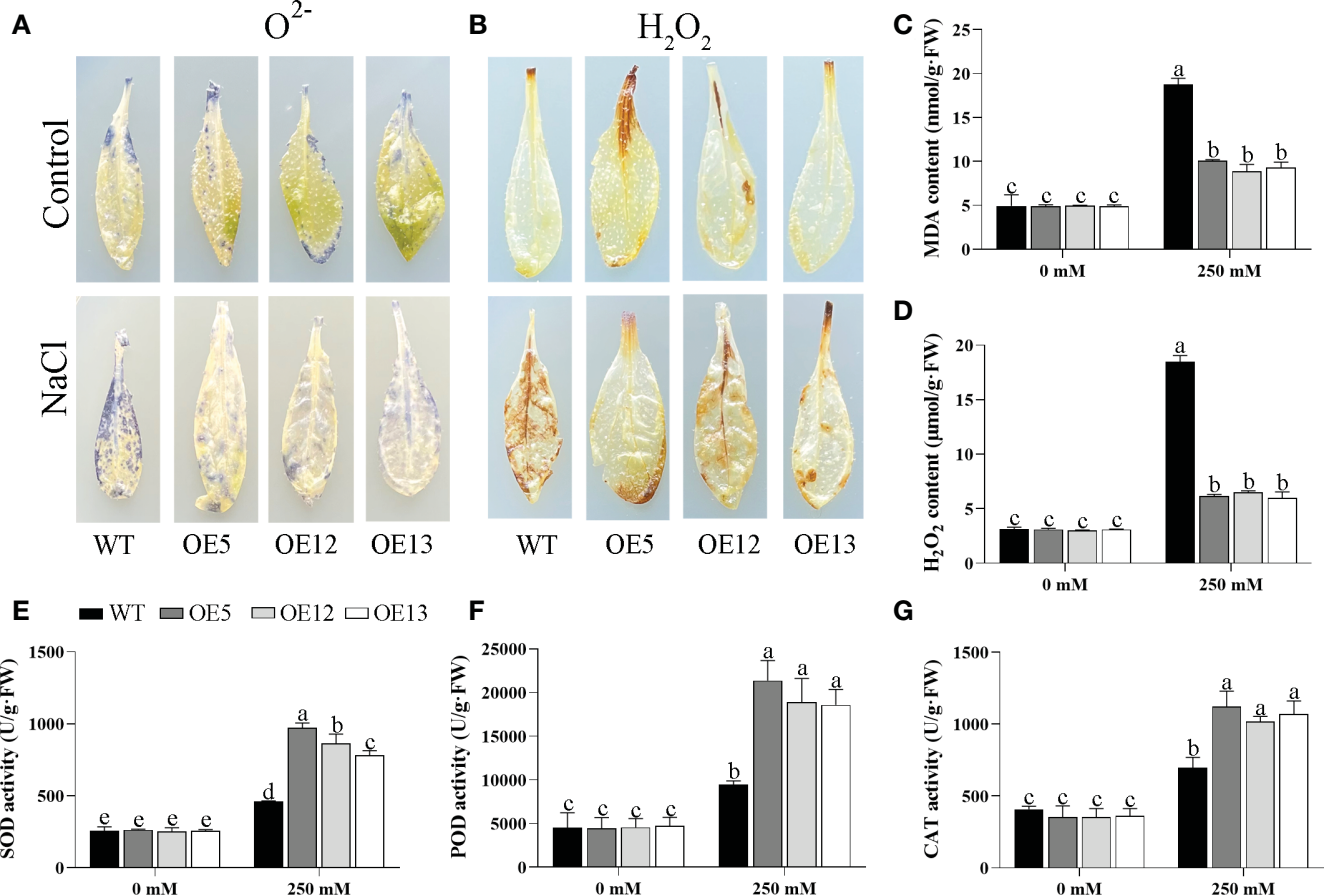
Changes in ROS accumulation and antioxidant enzyme activity in *CeqHAK6* transgenic lines and WT plants under salt treatment. **(A, B)** O^2-^ and H_2_O_2_ in the *CeqHAK6* transgenic lines and WT plants were detected by DAB and NBT staining. **(C, D, E, G)** MDA, H_2_O_2_, SOD, POD and CAT content were measured in WT and transgenic plants after salt treatment. All *Arabidopsis* seedlings were treated with 250 mM NaCl for 10 days. The significant differences identified using Duncan’s Multiple Range Test (*p*<0.05) were denoted by different lowercase letters on the top of bars.

**Figure 10:**
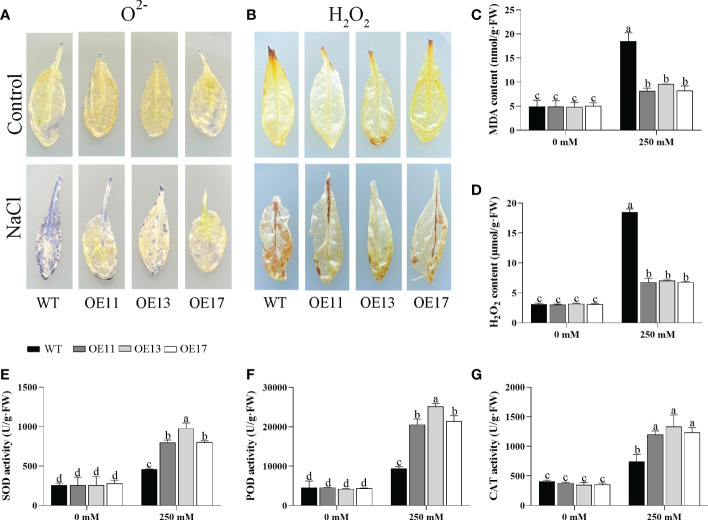
Changes in ROS accumulation and antioxidant enzyme activity in *CeqHAK11* transgenic lines and WT plants under salt treatment. **(A, B)** O^2-^ and H_2_O_2_ in the *CeqHAK11* transgenic lines and WT plants were detected by DAB and NBT staining. **(C, D, E, G)** MDA, H_2_O_2_, SOD, POD and CAT content were measured in WT and transgenic plants after salt treatment. All *Arabidopsis* seedlings were treated with 250 mM NaCl for 10 days. The significant differences identified using Duncan’s Multiple Range Test (*p*<0.05) were denoted by different lowercase letters on the top of bars.

The accumulation of hydrogen peroxide (H_2_O_2_) and superoxide ion (O^2-^) in leaves was detected by DAB and NBT staining under salt treatment. After salt stress, the leaves of the OE *CeqHAK6* lines showed lighter brown colour or blue spots than the WT (**Figures 9A, B**). Similarly, MDA and H_2_O_2_ contents of OE *CeqHAK6* lines were significantly lower than those of WT plants after salt treatment (**Figures 9C, D**). The reactive oxygen species (ROS) accumulation in *CeqHAK11* OE lines showed a similar trend to that in *CeqHAK6* (**Figures 10A–D**), implying that *CeqHAK6* and *CeqHAK11* alleviated the salt stress by accumulating less ROS in plant.

SOD, POD, and CAT activities were affected by salt stress. All three activities in Arabidopsis were significantly enhanced under salt stress (**Figures 9E–G**). The activities of these three enzymes in *CeqHAK6* OE and *CeqHAK11* OE lines were significantly higher than those in the (**Figures 10E–G**). The results demonstrated that *CeqHAK6* and *CeqHAK11* enhanced salt tolerance by increasing antioxidant enzyme activities and reducing ROS accumulation.

### *CeqHAK6* and *CeqHAK11* improved salt tolerance by maintaining high K^+^ in the leaves

Na^+^ and K^+^ concentrations in the WT and all OE lines were examined under NaCl treatment. Na^+^ content in leaves of *CeqHAK6* OE lines and WT increased slowly and did not differ substantially from 1 to 24 hours of salt treatment, while the accumulation of Na^+^ content in leaves of the OE lines was significantly lower than that of WT at 168 h (**Table 1**). Na^+^ concentration in leaves of *CeqHAK11* OE lines was marginally greater than that of WT at 24 h while Na^+^ content was markedly lower than that of WT at 168 h (**Table 2**). On the contrary, the K^+^ concentration in the leaves of all OE lines and WT plants decreased trend with increasing salt treatment time. However, the K^+^ content in the leaves of all OE lines maintained a relatively high level, and was twice more than that of the WT at 168 h salt treatment. This finding indicated that overexpression of *CeqHAK6* and *CeqHAK11* decreased Na^+^ content but increased K^+^ content in transgenic *Arabidopsis* leaves under salt stress, resulting in a high K^+^/Na^+^ ratio and improved transgenic *Arabidopsis* salt resistance.

**Table 1:** K^+^ and Na^+^ content in leaves of *CeqHAK6* overexpressing transgenic *Arabidopsis* and WT under different time periods of salt treatment.

Ion	OE Lines	0 h	1 h	6 h	24 h	168 h
K^+^ (g.kg^-1^)	WT	34.062 ± 1.127^a^	28.157 ± 1.292^fg^	23.233 ± 0.666^h^	19.028 ± 0.610^i^	5.693 ± 0.440^j^
	OE5	34.477 ± 0.5^a^	30.841 ± 0.516c^de^	29.964 ± 0.143^def^	27.631 ± 0.291^g^	22.887 ± 2.104^h^
	OE12	32.702 ± 2.275^abc^	31.71 ± 0.734^bcd^	29.907 ± 1^def^	27.945 ± 0.849^fg^	20.324 ± 1.369^i^
	OE13	33.553 ± 2.130^ab^	31.385 ± 0.408^cde^	29.528 ± 0.439^efg^	27.644 ± 0.994^g^	19.597 ± 1.510^j^
Na^+^ (g.kg^-1^)	WT	4.920 ± 0.479^ef^	6.705 ± 0.212^def^	7.379 ± 0.635^de^	8.616 ± 0.459^d^	81.181 ± 2.568^a^
	OE5	4.432 ± 0.276^ef^	5.065 ± 0.385^ef^	5.714 ± 0.322^def^	6.44 ± 0.426^def^	23.662 ± 3.068^c^
	vOE12	4.256 ± 0.262^ef^	4.651 ± 0.592^ef^	5.741 ± 0.340^def^	6.385 ± 0.493^def^	35.378 ± 3.284^b^
	OE13	3.814 ± 0.48^1f^	4.843 ± 0.192^ef^	5.225 ± 0.060^ef^	s6.265 ± 0.231^def^	37.081 ± 5.037^b^

The different letters represent significant differences among the treatments on each date (one-way analysis of variance (ANOVA) followed by using Duncan's Multiple Range Test, p<0.05).

**Table 2:** K^+^ and Na^+^ content in leaves of *CeqHAK11* overexpressing transgenic *Arabidopsis* and WT under different time periods of salt treatment.

Ion	OE Lines	0 h	1 h	6 h	24 h	168 h
K^+^ (g.kg^-1^)	WT	34.062 ± 1.127^a^	28.157 ± 1.292^cde^	23.233 ± 0.666^h^	19.028 ± 0.610^i^	5.693 ± 0.440^l^
	OE11	31.557 ± 2.130^b^	28.514 ± 0.408^cd^	25.876 ± 0.439e^fg^	23.436 ± 0.291^h^	13.041 ± 2.104^j^
	OE13	31.679 ± 1.744^b^	29.928 ± 0.087^bc^	27.043 ± 0.565^def^	24.297 ± 0.781^gh^	13.762 ± 2.307^j^
	OE17	32.04 ± 0.134^ab^	30.458 ± 0.737^bc^	27.181 ± 0.036^def^	24.892 ± 2.028^fgh^	9.799 ± 0.663^k^
Na^+^ (g.kg^-1^)	WT	4.920 ± 0.479^fg^	6.705 ± 0.212e^fg^	7.379 ± 0.635^ef^	8.616 ± 0.459^e^	81.181 ± 2.568^a^
	OE11	3.412 ± 0.231^g^	4.428 ± 0.181^fg^	5.885 ± 1.354^efg^	16.423 ± 2.829^d^	32.630 ± 3.591^b^
	OE13	3.901 ± 0.780f^g^	4.047 ± 0.214^fg^	5.122 ± 1.117^fg^	14.223 ± 1.609^d^	28.659 ± 4.556^c^
	OE17	4.238 ± 1.602f^g^	3.988 ± 0.396^fg^	5.827 ± 1.133e^fg^	15.138 ± 2.102^d^	30.418 ± 0.857b^c^

The different letters represent significant differences among the treatments on each date (one-way analysis of variance (ANOVA) followed by using Duncan's Multiple Range Test, p<0.05).

## Discussion

The maintenance of Na^+^/K^+^ balance in plants under salt stress is crucial for adaptation to adversity, and ion channel proteins and transporter proteins play an important role in ion uptake and transport (Li et al., 2018). HAK/KUP/KT transporter proteins have been widely characterized in *Arabidopsis* and rice, (Banuelos et al., 2002; Ahn et al., 2004; Cheng et al., 2018). However, HAK proteins have not been identified in woody plant, especially for *C. equisetifolia* which is being cultivated extensively for shelterbelts protection owing to its high salt tolerance.

### *CeqHAK6* and *CeqHAK11* genes were screened out based on systematic analysis of the HAK gene family

A total of 25 *HAK* genes were identified for *C. equisetifolia* in this stduy. Phylogenetic tree analysis showed that *CeqHAK* genes could be classified into four clusters (**Figure 1**), which was consistent with those described in previous stduies (Rubio et al., 2000; Gupta et al., 2008). *CeqHAK11* and its orthologous *OsHAK10*, *AtKUP6*, belonged to cluster II, and the members of this family were different in sequence and function. For example, *AtKUP1* mediates both high- and low-affinity K^+^ transporters, while *OsHAK10* has low-affinity K^+^ uptake properties (Kim et al., 1998; Banuelos et al., 2002). A close evolutionary relationship exists between subgroups, as evidenced by the fact that the most conserved motifs across members of the same subgroup shared certain similar features. However, the presence of different types of motif indicated potential diversity of function. It has been shown that many members of cluster I can respond positively to low potassium stress, such as *OsHAK1*, *OsHAK5*, and *AtHAK5* (Banuelos et al., 2002; Pyo et al., 2010). It offers a theoretical foundation for additional research into the biological roles of the HAK gene family in *C. equisetifolia* by combining bioinformatics analysis with transcriptome data.

The accumulated studies revealed that most of the HAK/KUP/KT family members are highly expressed primarily in the roots of plants, while some members are highly expressed in the whole plant (Osakabe et al., 2013; Shen et al., 2015; Han et al., 2016; Chen et al., 2018). In the present stduy, *CeqHAK6* was highly expressed in the roots and stems, whereas *CeqHAK11* was highly expressed mainly in the xylem and phloem of stems (**Figure 3**). The plasma membrane localization of *CeqHAK6* and *CeqHAK11* (**Figure 4**) was similar to that of maize *ZmHAK4*. In addition, *ZmHAK4* mainly promoted shoot Na^+^ exclusion for improving salt tolerance (Zhang et al., 2019). The expression level of *CeqHAK11* and *CeqHAK6* increased significantly with the duration of salt treatment. All these results implied that *CeqHAK11* and *CeqHAK6* might play a key role in the salt tolerance mechanism of *C. equisetifolia*.

### *CeqHAK6* and *CeqHAK11* were putative essential genes for K^+^ acquisition and translocation

Mounting evidence indicates that cluster I HAKs confer activity of high-affinity K^+^ uptake, and their transcript levels increase under low-K^+^ conditions (Yang et al., 2014; Shen et al., 2015; Li et al., 2018). For example, *OsHAK1* and *OsHAK5* mediate high affinity absorption of K^+^ in the presence of low external K^+^ content (Chen et al., 2018). In a recent study, cereal (*Setaria italica*) *SiHAK1* expression was significantly up-regulated in a low-potassium environment and promoted plant K^+^ uptake. Furthermore, it was found that *SiHAK1* had better potassium transport capacity than *OsHAK1*, *OsHAK5* and *HvHAK1* under low potassium environment (Zhang et al., 2018). In the present study, we present clear evidence that HAK transporter *CeqHAK6* in cluster I was a high-affinity K^+^ transporter. Interestingly, the capability of *CeqHAK11* to mediate high affinity K^+^ uptake is stronger than the *CeqHAK6* (**Figure 5**). Similarly, *ZmHAK1* and *ZmHAK5* transformants recover the growth at K^+^ deficiency, but *ZmHAK1* is less potent for potassium transport than *ZmHAK5* (Qin et al., 2019).

It is well known that about 40-90% of K^+^ absorbed in plants roots could be re-transported from the shoot through phloem and recycled in the roots (Li et al., 2018). However, in comparison to K^+^ uptake, there has been little research into the role of HAK transporters in K^+^ upward translocation from roots to shoots. *OsHAK5* was highly expressed in xylem parenchyma and phloem of root, and participated in the upward transport of K^+^ from the root to aerial parts (Yang et al., 2014). Similarly, *AtKUP7* may be involved in such long-distance transport of K^+^, especially under low-K^+^ conditions (Han et al., 2016). In this study, *CeqHAK6* was highly expressed in the root and the xylem and phloem of the stem while *CeqHAK11* was highly expressed in the phloem of the stem. After treating *Arabidopsis* seedlings with 200 mM NaCl for 168 h, the K^+^ content in the leaves of all OE lines was significantly increased compared to the WT. Therefore, *C. equisetifolia HAK6* and *HAK11* are high-affinity transporters of K^+^ uptake and might be implicated in the long-distance transport of K^+^.

### *CeqHAK6* and *CeqHAK11* genes improved plant salt tolerance by preserving the K^+^/Na^+^ balance and lowering ROS generation

The accumulated research data suggested that the yeast expressing *HAKs* grew better than the control in high salt environment and had obvious Na^+^ resistance (Guo et al., 2008; Okada et al., 2018). Our results are consistent with this information in that *CeqHAK6* and *CeqHAK11* enhanced the salt tolerance of yeast strain. In rice, *OsHAK16* also improved the salt tolerance of plants (Feng et al., 2019). In reed, *PhaHAK1* and *PhaHAK2* also enhanced salt tolerance, which mainly mediated K^+^ uptake and maintained K^+^/Na^+^ ratios (Takahashi et al., 2007b; Takahashi et al., 2007a). In the present study, *CeqHAK6* OE and *CeqHAK11* OE lines had higher germination and better root growth under salt treatment (**Figures 7**, **8**). Furthermore, *CeqHAK6* and *CeqHAK11* transgenic seedlings demonstrated improved high salt tolerance under 250 mM NaCl treatment (**Figure 6**). Consistent with the phenotypic results, the K^+^ content in the leaves of all OE lines increased significantly whie Na^+^ declined dramatically at 168 h of salt treatment compared to the WT. Therefore, overexpression of *CeqHAK6* and *CeqHAK11* improves salt tolerance of plants by maintaining a higher K^+^/Na^+^ ratio in the plants.

It is well known that reactive oxygen species (ROS) are produced in plants under salt stress, and excessive accumulation of ROS can cause oxidative damage to membranes, proteins and nucleic acids thereby disrupting normal physiological metabolism (Mittova et al., 2002; Suzuki et al., 2012). For instance, *DcWRKY3* OE lines accumulated less ROS than WT leaves under salt stress, and *DcWRKY3* has been implicated in improving the salt tolerance of plants (Yu et al., 2022). Similarly, the WT leaves accumulated more O^2-^ and H_2_O_2_ than OE lines of *CeqHAK6* and *CeqHAK11* under salt stress by NBT and DAB staining. Correspondingly, the content of MDA and H_2_O_2_ in *CeqHAK6* OE and *CeqHAK11* OE lines decreased significantly under salt treatment (**Figures 9**, **10**). Some previous studies have indicated that ROS scavenging systems are essential to prevent oxidative damage in plants under stresses (Zhu, 2001; Xing et al., 2022). In rice, overexpression of *OsHAK1* had lower level of lipid peroxidation, and higher activities of antioxidant enzymes (POX and CAT), indicating that *OsHAK1* gene enhances plant drought resistance by reducing ROS accumulation (Chen et al., 2018). Our results demonstrated that the higher activities of CAT, POD and SOD in *CeqHAK6* and *CeqHAK11* OE lines were necessary to improve plant salt tolerance of plants.

An earlier study by Li et al. (2022) revealed that high salt tolerance was conferred by the synergistic contribution of ROS scavenging mechanisms and Na^+^/K^+^ transport to redox balance and ion homeostasis. Overexpression of *CeqHAK6* and *CeqHAK11* increased the K^+^/Na^+^ ratio and antioxidant enzyme activity of plants, which in turn reduced the accumulation of malondialdehyde, cations and H_2_O_2_ due to membrane peroxidation, and finally achieved the purpose of protecting membrane structure and alleviating the damage caused by salt stress to the plants. In addition, *C. equisetifolia* lacks stable and efficient genetic transformation system, so this study hopes to use salt tolerance genes characterized in *C. equisetifolia* for genetic manipulation to improve development of salt tolerance crop varieties.

## Conclusion

This study identified 25 *HAK* genes in *C. equisetifolia* and further analysis showed that *CeqHAK11* and *CeqHAK6* could constantly respond to salt stress. *CeqHAK6* and *CeqHAK11* genes were located in the plasma membrane and mediated K^+^ absorption and transport. Moreover, the *CeqHAK6* and *CeqHAK11* genes improved salt tolerance of plants by maintaining the K^+^/Na^+^ balance, increasing antioxidant enzyme activity and lowering ROS generation. Overall, this study provides a solid theoretical foundation for salt tolerance research and breeding for salt tolerance of *C. equisetifolia*.

## Data availability statement

The datasets presented in this study can be found in online repositories. The names of the repository/repositories and accession number(s) can be found in the article/**Supplementary Material**.

## Author contributions

YJW: Conceptualization, Writing- Original draft, Writing- Review and Editing. YZ, CZ: Funding acquisition, Writing- Review and Editing. CF: Conceptualization, Supervision, Writing- Review and Editing. YCW, JM: Formal analysis, Software, Visualization. All authors contributed to the article and approved the submitted version.
