# Supplementary material for: Characterization of HAK protein family in Casuarina equisetifolia and the positive regulatory role of CeqHAK6 and CeqHAK11 genes in response to salt tolerance
Source: Front Plant Sci. 2023 Feb 3;13:1084337. doi: 10.3389/fpls.2022.1084337 (PMC9936244; doi:10.3389/fpls.2022.1084337)
Supplement: Supplementary file 1 [file Table_1.docx]

**Characterization of HAK Protein Family in *Casuarina equisetifolia* and the Positive**

**Regulatory Role of *CeqHAK6* and *CeqHAK11* in Response to Salt Tolerance**

Yujiao Wang^1^, Yong Zhang^1*^, Yongcheng Wei^1^, Jingxiang Meng^1^, Chonglu Zhong^1^, Chunjie Fan^1*^

^1^ State Key Laboratory of Tree Genetics and Breeding, Key Laboratory of State Forestry and Grassland Administration on Tropical Forestry, Research Institute of Tropical Forestry, Chinese Academy of Forestry, Guangzhou 510520, China

* Corresponding author e-mail:

**zhangyongritf@caf.ac.cn;**

**zclritf@126.com**

**fanchunjie@caf.ac.cn;**

**Supplementary figures**


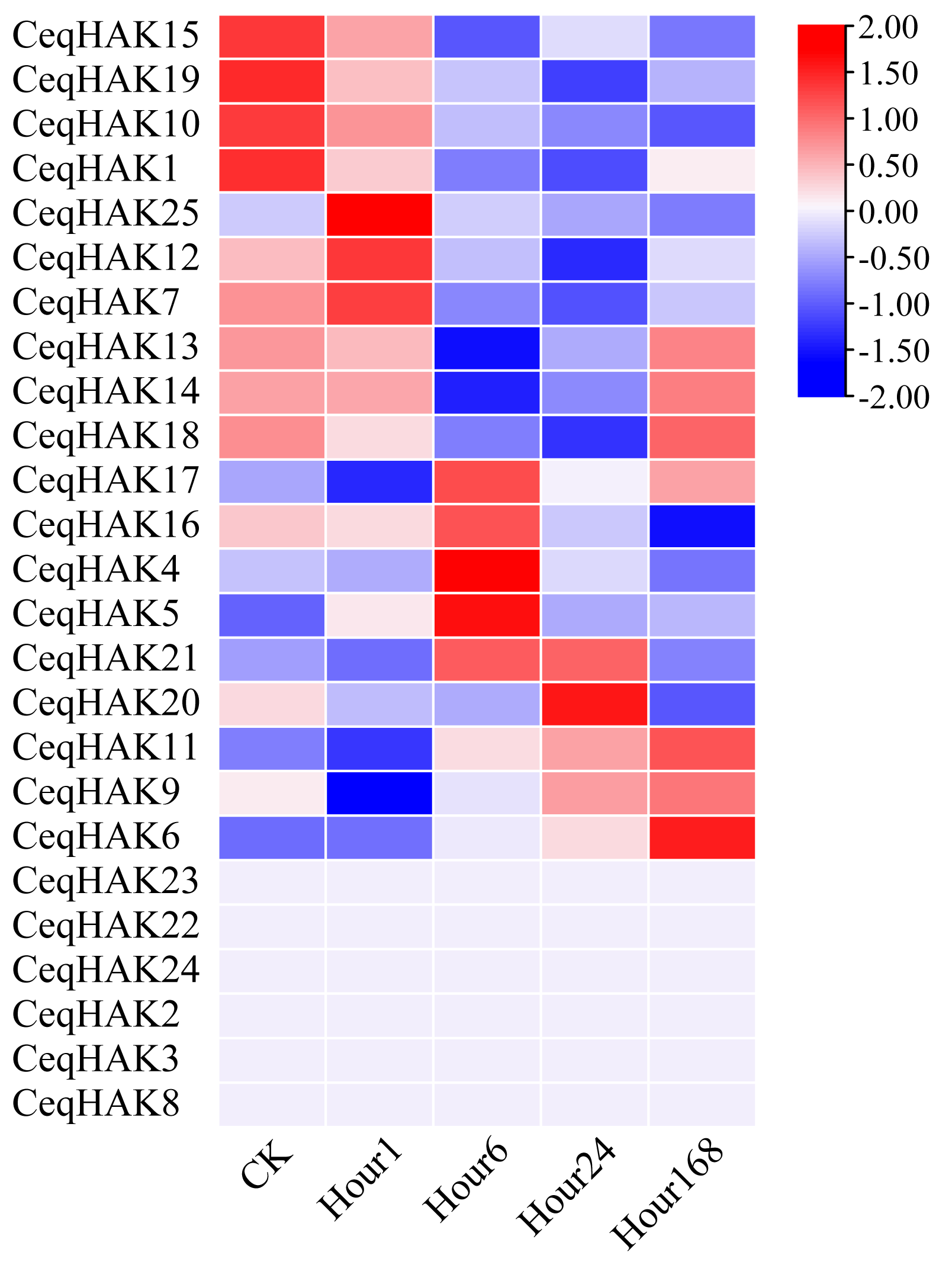


**Supplementary Figure S1.** **Expression pattern of 25 *CeqHAK* genes following NaCl treatment at different time points as determined by RNA-Seq.** The color scale represents log10 expression values, blue represents low expression and red indicates a high expression level (transcript abundance).


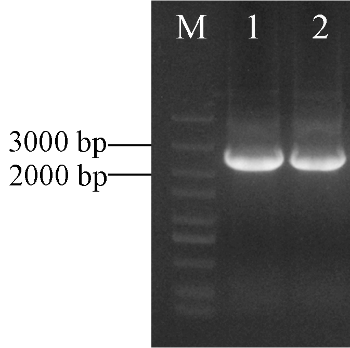


**Supplementary Figure S2. PCR amplified product of *CeqHAK6* and *CeqHAK11* genes.** M: DNA marker; 1: PCR amplified product of the *CeqHAK11*; 2: PCR amplified product of the *CeqHAK6*.


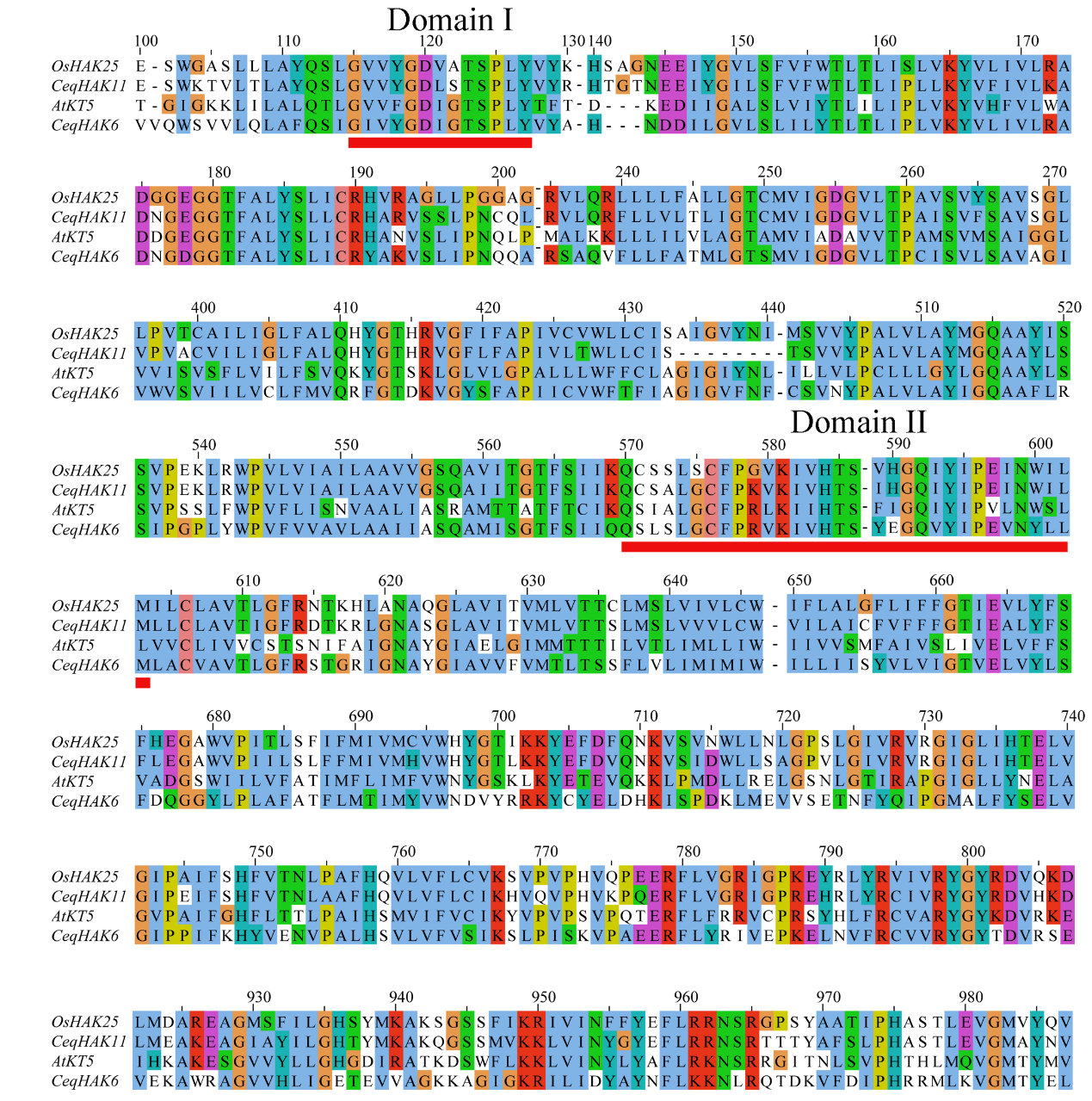


**Supplementary Figure S3.** **Multiple sequence alignment of CeqHAK6, CeqHAK11, AtKT5 and OsHAK25 proteins.** Multiple sequence alignment was performed using ClustalX 2.11.


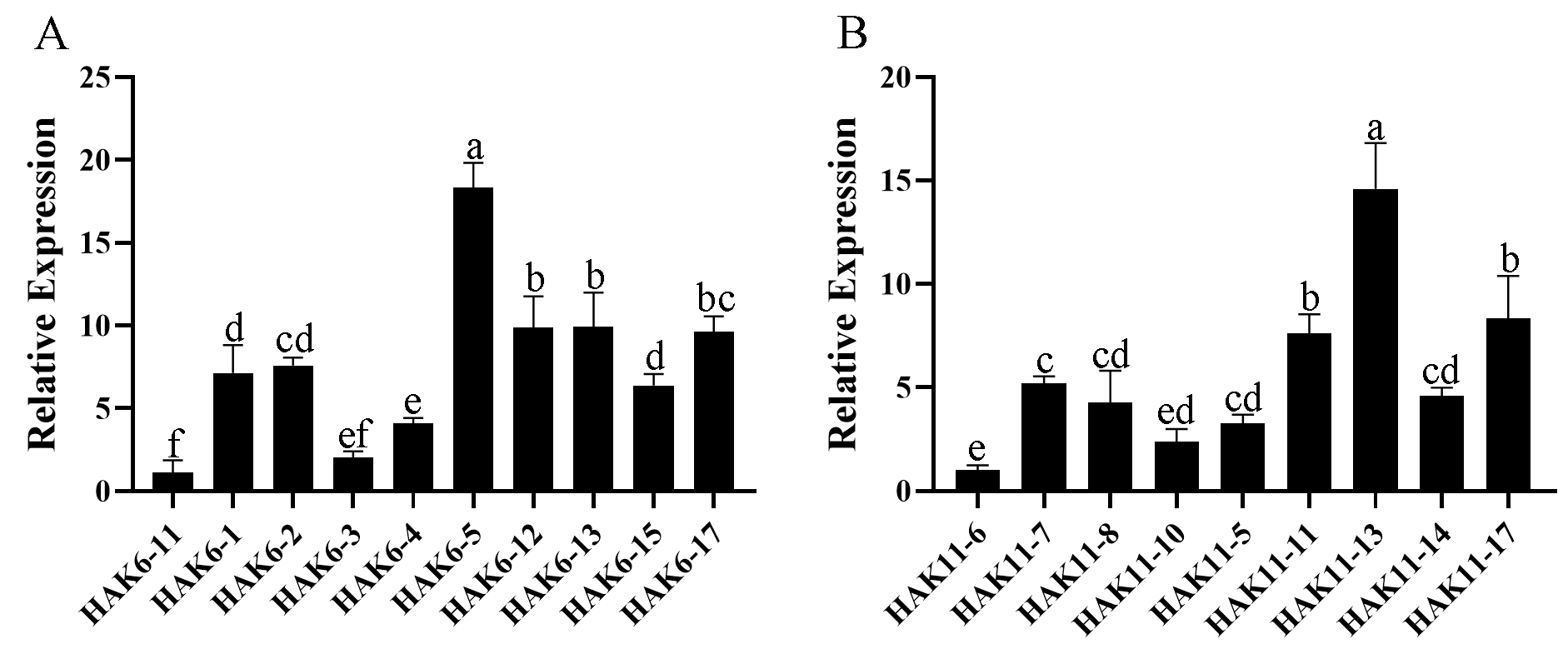


**Supplementary Figure S4.** **The expression levels of *CeqHAK6* and *CeqHAK11* transgenic lines were detected by qPCR analysis.** Mean values and standard deviations (SDs) were obtained from three biological and three technical replicates. The significant differences identified using Duncan's Multiple Range Test (p<0.05) were denoted by different lowercase letters on the top of bars.
